# Differentially methylated genes involved in reproduction and ploidy levels in recent diploidized and tetraploidized *Eragrostis curvula* genotypes

**DOI:** 10.1007/s00497-023-00490-7

**Published:** 2023-12-06

**Authors:** J. Carballo, A. Achilli, F. Hernández, M. Bocchini, M. C. Pasten, G. Marconi, E. Albertini, D. Zappacosta, V. Echenique

**Affiliations:** 1 Centro de Recursos Naturales Renovables de la Zona Semiárida (CERZOS–CCT–CONICET Bahía Blanca), Camino de La Carrindanga Km 7, 8000 Bahía Blanca, Argentina; 2 Departamento de Agronomía, Universidad Nacional del Sur (UNS), San Andrés 800, 8000 Bahía Blanca, Argentina; 3 Dipartimento di Scienze Agrarie, Alimentari e Ambientali, Università degli Studi di Perugia, 06121 Perugia, Italy

**Keywords:** Methylation, Apomixis, Epigenetics, Eragrostis curvula

## Abstract

Epigenetics studies changes in gene activity without changes in the DNA sequence. Methylation is an epigenetic mechanism important in many pathways, such as biotic and abiotic stresses, cell division, and reproduction. *Eragrostis curvula* is a grass species reproducing by apomixis, a clonal reproduction by seeds. This work employed the MCSeEd technique to identify deferentially methylated positions, regions, and genes in the CG, CHG, and CHH contexts in *E*. *curvula* genotypes with similar genomic backgrounds but with different reproductive modes and ploidy levels. In this way, we focused the analysis on the cvs. Tanganyika INTA (4x, apomictic), Victoria (2x, sexual), and Bahiense (4x, apomictic). Victoria was obtained from the diploidization of Tanganyika INTA, while Bahiense was produced from the tetraploidization of Victoria. This study showed that polyploid/apomictic genotypes had more differentially methylated positions and regions than the diploid sexual ones. Interestingly, it was possible to observe fewer differentially methylated positions and regions in CG than in the other contexts, meaning CG methylation is conserved across the genotypes regardless of the ploidy level and reproductive mode. In the comparisons between sexual and apomictic genotypes, we identified differentially methylated genes involved in the reproductive pathways, specifically in meiosis, cell division, and fertilization. Another interesting observation was that several differentially methylated genes between the diploid and the original tetraploid genotype recovered their methylation status after tetraploidization, suggesting that methylation is an important mechanism involved in reproduction and ploidy changes.

**Supplementary Information:**

The online version contains supplementary material available at 10.1007/s00497-023-00490-7.

## Introduction

Epigenetics studies heritable gene expression changes without altering the DNA sequence (Russo 1996; Armstrong 2013). The main epigenetic mechanisms are DNA methylation, histone modifications, RNA interference, and genomic imprinting (Munshi et al. 2015). Methylation occurs when a methyl group is added to a cytosine in the 5′ position in three contexts: CG, CHH, and CHG where H represents an A, C, or T (Chen and Li 2004). Methylation is involved in gene regulation and genome stability and has an important role in many pathways, such as seed imprinting, response to biotic and abiotic stresses, and cell division (Zhang et al. 2018). More specifically, methylation and de-methylation dynamics during reproduction are fundamental for normal plant propagation (Melamed-Bessudo and Levy 2012; Underwood et al. 2018; Han et al. 2019).

One of the most widely used methods to assess DNA methylation is whole-genome bisulfite sequencing (Lister et al. 2008). However, this method requires extraordinary coverage, increasing the costs significantly. The Methylation Context Sensitive Enzyme ddRAD (MCSeEd) technique was recently developed to reduce sequencing costs (Marconi et al. 2019; Di Marsico et al. 2020). This method was successfully used to study methylation changes associated with different plant growth conditions, such as drought in *Zea maize* (Marconi et al. 2019), asexual reproduction of *Eragrostis curvula* and *Paspalum rufum* (Carballo et al. 2021a; Soliman et al. 2021), response to infection with the cereal pathogen *Fusarium graminearum* (Tini et al. 2021), and the chilling accumulation period in *Prunus persica* (Canton et al. 2022).

**Fig. 1:**
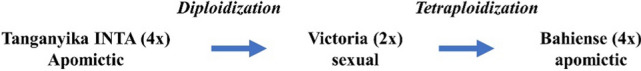
Diagram representing the recent events of diploidization of Tanganyika INTA to obtain Victoria and the tetraploidization of Victoria to obtain Bahiense

*Eragrostis curvula* is a C4 grass species used as forage in semiarid regions. This species is primarily investigated because of its capability to reproduce by apomixis. (Carballo et al. 2021b). Apomixis is an asexual reproduction by seeds in which the progeny is genetically identical to the maternal plant. Three main components differentiate apomixis from sexual reproduction: apomeiosis, parthenogenesis, and pseudogamy (Crane 2001). During the *E. curvula* embryo sac development, the meiosis is completely absent (apomeiosis), the embryo arises without fertilization (parthenogenesis), and only the polar nuclei are fertilized (pseudogamy). One advantage of using *E. curvula* as a model species for apomixis is that sexual and apomictic genotypes co-exist in this species. Even more, a synthetic line with a similar genetic background was created to study changes in reproductive mode and ploidy level (Cardone et al. 2006). In this way, the sexual diploid Victoria was obtained from in vitro culture of inflorescences of the apomictic tetraploid Tanganyika INTA cultivar, and the apomictic tetraploid Bahiense was obtained from the polyploidization of Victoria (Fig. 1). It is currently accepted that all seed plants have experienced at least one round of whole genome duplication in their evolutionary history (Jiao et al. 2011). After this, many species recover their diploid level due to the loss of genes during evolution. In *E. curvula*, the synthetic diploidization followed by polyploidization is considered a recent event compared with the ancestral duplication. This synthetic line is an ideal tool to compare genotypes with different ploidy levels and reproductive modes but with similar genomic background.

Albeit the genetic and epigenetic regulation of apomixis in *E. curvula* is not completely clear, several advances have been made in the last few years. Using genomics and transcriptomics approaches over sexual and apomictic genotypes, different candidate genes and regulatory pathways were found to be differentially enriched (Garbus et al. 2017; Carballo et al. 2019, 2023; Zappacosta et al. 2019; Selva et al. 2020). In addition, analyses of methylation patterns and microRNA representation in different genotypes shed light on the epigenetic regulation of apomixis (Rodrigo et al. 2017; Garbus et al. 2019; Carballo et al. 2021a; Pasten et al. 2022). Methylation studies using the MSAP technique were used in tetraploidized and diploidized lines, showing lower methylation levels in the diploid genotypes and methylation recovery after tetraploidization (Ochogavia et al. 2009). However, this technique cannot identify differentially methylated genes. The same technique in facultative apomictic *E. curvula* plants exposed to stress showed a positive correlation between DNA methylation changes and the percentage of sexual reproduction (Rodrigo et al. 2017). These results suggest a quantitative regulation of apomeiosis in facultative cultivars mediated by methylation under stress conditions. More recently, we reported higher levels of methylation in *E. curvula* tetraploid apomictic genotypes than in the sexual one with the same ploidy level (Carballo et al. 2021a). In the mentioned study, genes related to reproductive pathways, like the ubiquitin one, were differentially methylated between genotypes. However, the genetic background of the analyzed genotypes was completely different, not allowing us to distinguish whether the differences could be attributed to the reproductive mode or were associated with differences in the genotypes themselves.

Here, we employed the MCSeEd method to study methylation changes across synthetically developed *E. curvula* genotypes with similar genomic background but with different reproductive modes and ploidy levels. This work aimed to identify differentially methylated genes and pathways associated with reproduction and ploidy in the recent diploidized and tetraploidized *E. curvula* genotypes.

## Materials and methods

### Plant material and DNA extraction

The genotypes used for methylation analyses were the apomictic Tanganyika INTA (2n = 4X = 40), Bahiense (2n = 4X = 40), and TUNS9355 (2n = 6X = 60) cultivars, and the sexual genotype Victoria (2n = 2X = 20). Tanganyika INTA, Victoria, and Bahiense were chosen based on their genomic background (Fig. 1), and TUNS9355 was selected in order to incorporate an unrelated polyploid apomictic genotype. The most plausible explanation for the emergence of the diploid genotype is that a Tanganyika INTA unreduced gamete regenerates, producing Victoria. Samples were collected from inflorescences, including stages from archespore to mature embryo sac, in order to target both apomeiosis and parthenogenesis. Three biological replicates were taken from each sample from plants growing at a CERZOS (CONICET-UNS) greenhouse (Bahía Blanca, Argentina; 38°42′ S, 62°16′ W). Genomic DNA was extracted using a cetyltrimethylammonium bromide protocol. Briefly, spikelets were frozen and ground in liquid nitrogen to obtain a fine powder by using a TissueLyser II (Qiagen). Then, 100 mg from each sample was incubated at 65 °C in preheated extraction buffer containing 100 mM Tris HCl pH 8, 1.4 M NaCl, 20 mM, EDTA pH 8, 2% (w/v) CTAB, and 0.5% (v/v) β-mercaptoethanol. Chloroform was added to reach a 2:1 ratio (buffer/chloroform), and the aqueous phase was collected after centrifugation. DNA was precipitated with one volume of isopropanol and washed with 70% (v/v) ethanol two times. Finally, the pellet was air-dried and resuspended in 50 µL of Milli-Q water containing 20 µg/mL RNase.

### Library preparation

Following the MCSeEd protocol (Marconi et al. 2019; Di Marsico et al. 2020), each sample consisting of 150 ng of DNA was treated with the *Mse*I enzyme to decrease genome complexity and with the methylation-sensitive enzymes *Aci*I, *Pst*I*,* and *EcoT22*I to detect methylation over the CG, CHG, and CHH contexts, respectively. After the double digestion, specific barcodes were ligated to demultiplex the samples in the downstream analysis. Then, samples were sequenced using the Illumina Hiseq X platform (Table S1). Finally, demultiplexing was carried out using the process_radtags script (Catchen et al. 2013). Reads were uploaded to NCBI database under the BioProject: PRJNA988943.

### Bioinformatic analysis

Bioinformatic analysis followed the MCSeEd reference pipeline (Marconi et al. 2019, https://bitbucket.org/capemaster/mcseed/src/master/). After demultiplexing, the reads were mapped against the *E. curvula* Victoria genome assembly (Carballo et al. 2019), obtaining a minimum of 80% and a maximum of 97% of the mapping rate. Reads per position were normalized, and the differentially methylated positions (DMPs) were detected by using the methylkit R package (Akalin et al. 2012). As described by Marconi et al. (2019), the differentially methylated regions (DMRs) were obtained by identifying the best window for each context in which two or more positions had the same behavior (i.e., de-methylated or methylated). Differentially methylated genes (DMGs) were detected by contrasting the position of the DMRs with the Victoria genome annotation (Carballo et al. 2019). The DMGs analysis focused on the gene body and 2500 bp before and after the start and stop codons for the upstream and downstream regions, respectively. This window was chosen because in plants, gene expression regulation via methylation was previously associated with the promoter and enhancer regions located up to 2500 bp upstream and downstream, respectively (He et al. 2020, 2022; Bewick and Schmitz 2017). DMPs, DMRs, and DMGs comparisons were performed in all versus all plant materials, as follows: Victoria versus Bahiense, Victoria versus Tanganyika INTA, Victoria versus TUNS9355, Bahiense versus Tanganyika INTA, Bahiense versus TUNS9355, and Tanganyika INTA versus TUNS9355, hereafter referred as vicVSbah, vicVStan, vicVSt93, bahVStan, bahVSt93, and tanVSt93 respectively.

### Differentially methylated genes

To distinguish DMGs related to the reproductive mode, the comparisons between apomictic versus sexual genotypes were specially analyzed, considering those DMGs with the same behavior in the three contrasts (vicVSbah, vicVStan, and vicVSt93). On the other hand, to detect the methylation changes related to diploidization and tetraploidization, the comparisons vicVStan and vicVSbah were also analyzed. In these comparisons, methylation and de-methylation always refer to the first genotype; for instance, in vicVStan, methylated and de-methylated DMPs, DMRs, and DMGs refer to Victoria. The genes were annotated using the string database (Szklarczyk et al. 2019). Differentially gene ontology analyses were performed using the clusterProfiler R package (Yu et al. 2012).

## Results

### Differentially methylated positions and regions

DNA from inflorescences of *E. curvula* genotypes Tanganyika INTA, Victoria, Bahiense, and TUNS9355 were extracted and sequenced in three biological replicates following the MCSeEd protocol to determine the methylation changes in CG, CHG and CHH contexts (Marconi et al. 2019). For this purpose, the Victoria genome assembly (Carballo et al. 2019) was used as a reference to identify differentially methylated positions (DMPs), differentially methylated regions (DMRs), and differentially methylated genes (DMGs). Even though Tanganyika INTA and Bahiense are facultative apomictic tetraploids and Victoria is a sexual diploid, they share a similar genomic background since Victoria was obtained from inflorescences of Tanganyika INTA through in vitro culture, and Bahiense derives from Victoria (Fig. 1) (Cardone et al. 2006). The hexaploid facultative apomict TUNS9355 was also included in the analysis to evaluate the effect of methylation in apomictic genotypes unrelated to the tetraploidized and diploidized genotypes.

By employing the methylkit pipeline (Akalin et al. 2012), a total of 42,780, 46,132, and 53,707 methylated/de-methylated positions were estimated in the Victoria genome assembly for the CG, CHG, and CHH contexts, respectively. The principal component analysis performed with these data showed a high similarity within biological replicates and differences between genotypes, thus evidencing data suitability for the downstream analysis (Figure S1). Based on the phylogenetic tree, there was no evident clustering in common for all three contexts since Victoria, TUNS9355, and Bahiense grouped for methylations/de-methylations in the CG context, while in CHG context Tanganyika INTA, Victoria, and Bahiense clustered together. Finally, for CHG context, Victoria grouped with Bahiense, and Tanganyika with TUNS9355 (Figure S2).

**Fig. 2:**
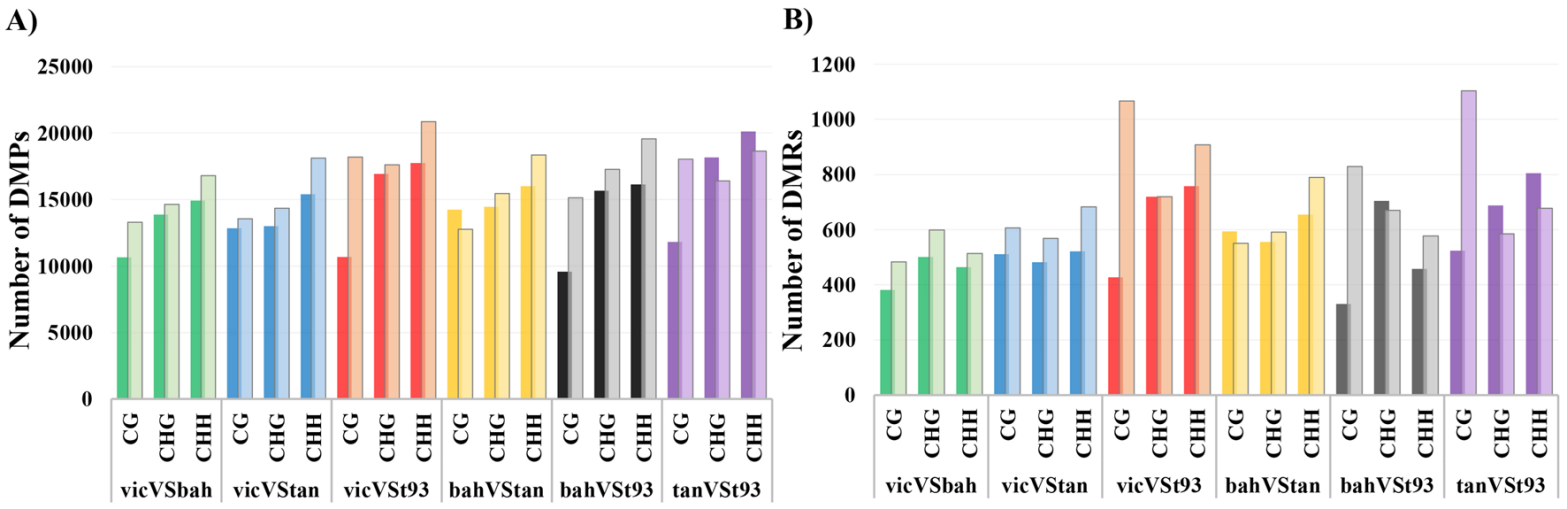
Number of differentially methylated and de-methylated positions (A) and regions (B) for the comparisons: Victoria versus Bahiense (vicVSbah), Victoria versus Tanganyika INTA (vicVStan), Victoria versus TUNS9355 (vicVSt93), Bahiense versus Tanganyika INTA (bahVStan), Bahiense versus TUNS9355 (bahVSt93), and Tanganyika INTA versus TUNS9355 (tanVSt93). Full-color bars represent methylated and transparent de-methylated positions/regions

DMPs and DMRs were computed in all comparison combinations: vicVSbah, vicVStan, vicVSt93, bahVStan, bahVSt93, and tanVSt93 (Fig. 2). In these comparisons, the context with higher DMPs was CHH, followed by CHG and CG (Fig. 2, Table S2). This distribution was not reflected on DMRs since this relationship was only retained for vicVStan and bahVStan (Fig. 2, Table S3). The DMPs within DMRs were plotted in a heatmap for all the analyses, showing a high correlation between replicates and the expected differences between genotypes (Figure S3).

In the comparisons between sexual and apomictic genotypes (i.e., vicVSbah, vicVStan, and vicVSt93), which is coincident with diploid versus polyploid genotypes, it was possible to observe more de-methylated DMPs and DMRs in the sexual genotype for all the contexts (Fig. 2). Tetraploid versus tetraploid contrasts (bahVStan) showed in Bahiense more methylated DMPs and DMRs in CG contexts, while in CHG and CHH, the number of de-methylated DMPs and DMRs was higher than Tanganyika INTA cultivar. The hexaploid versus tetraploid analysis (bahVSt93 and tanVSt93) displayed differences in DMPs and DMRs associated with the contexts. The study of DMPs in bahVSt93 showed that de-methylation in Bahiense was higher in all the contexts. The same analysis over DMRs showed more methylation in Bahiense in the CHG context. On the other hand, in the tanVSt93 comparison, the de-methylation in Tanganyika INTA was higher in CG and lower in CHG and CHH contexts in both DMPs and DMRs.

**Fig. 3:**
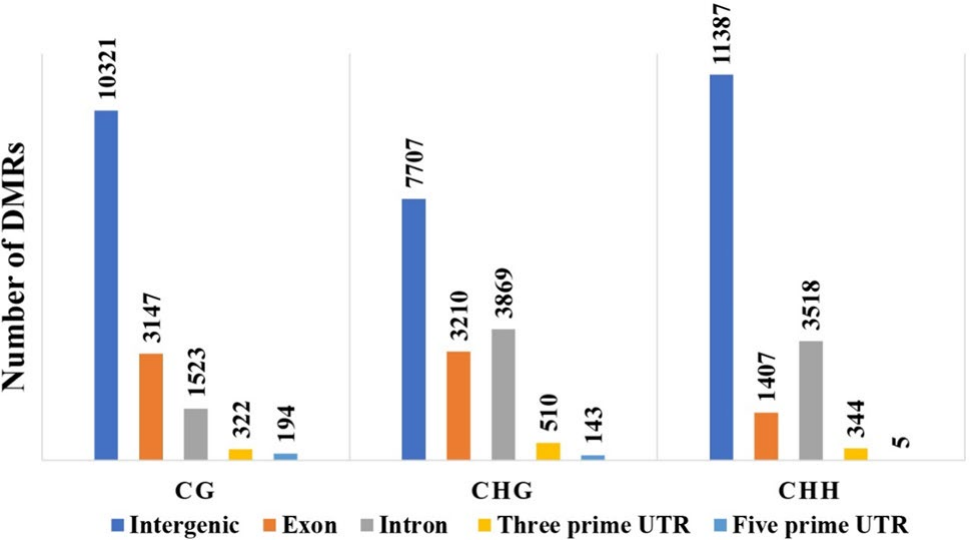
Number of DMRs found within intergenic, introns, exons, and UTR regions in the Victoria genome

When the positions of the DMR were contrasted with the Victoria genome, it was possible to observe an increased number of DMRs within intergenic areas in the CG, CHG and CHH contexts (Fig. 3). The number of DMRs in the 3´ UTR was higher than those in the 5´ UTR in all three contexts. The number of DMRs in introns and exons was linked to the methylation context; in CG, DMRs were more present in exons, while introns had a higher number of DMRs for the CHG and CHH contexts.

### Differentially methylated genes

**Fig. 4:**
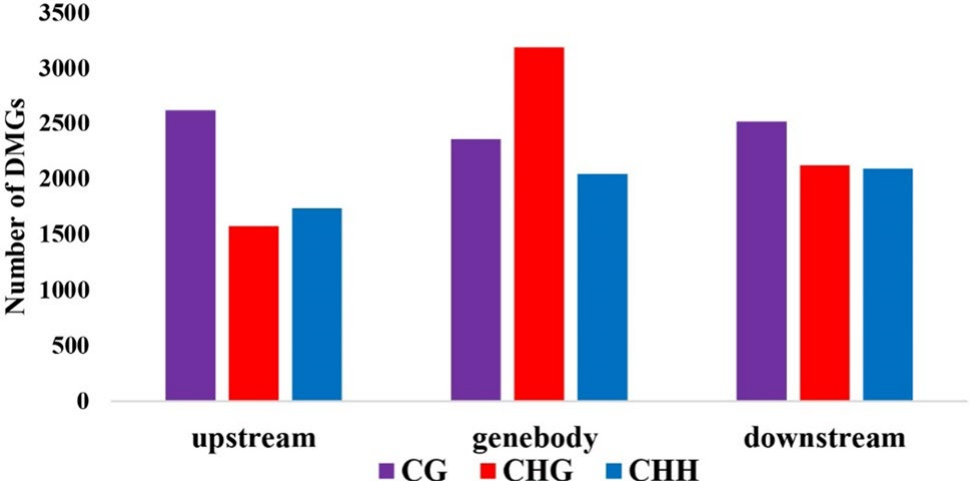
Number of differentially methylated genes in CG, CHG, and CHH contexts in upstream, downstream, and gene body regions

DMRs within genes in the CG, CHG, and CHH contexts were analyzed for the vicVSbah, vicVStan, vicVSt93, bahVStan, bahVSt93, and tanVSt93. Since the regulation of gene expression is associated with the location of methylated positions within the gene, DMGs were classified according to whether the methylated position was located upstream, downstream or within the gene body (Table S4). Even though the total number of DMGs was similar across the upstream, gene body, and downstream regions, the distribution within each region was associated with the context. In this way, the number of DMGs in CG and CHH contexts was similar in the three regions, while the number of DMGs in the CHG context was higher in the gene body than upstream and downstream (Fig. 4). The number of methylated and de-methylated positions within DMGs was similar in all the samples in the three regions. There were no significant differences, with a minimum of 2 and a maximum of 2.37 methylated positions on average (Table S5).

**Fig. 5:**
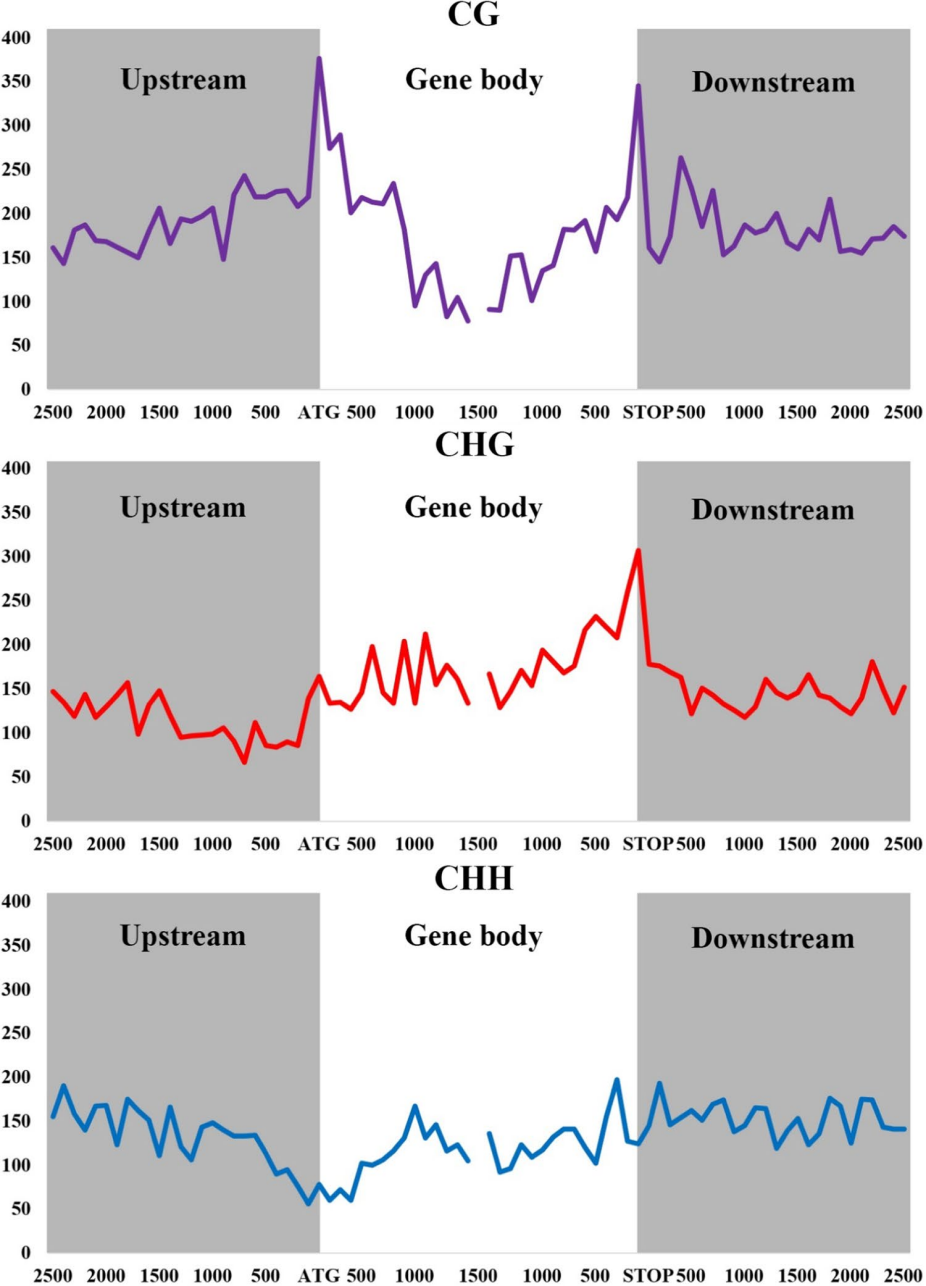
DMRs in the CG, CHG, and CHH contexts across the upstream, gene body, and downstream regions

The distribution of DMRs across the genes was computed considering 2,500 bp upstream and downstream from the start and stop position, respectively, and 1,000 bp after and before the start and stop position, respectively (Fig. 5). The distribution of DMRs in the three contexts displayed different patterns. The highest number of DMRs in the CG context was found in the area surrounding the start and stop positions, with a peak of ~ 350 DMRs, respectively. In the CHG context, the stop codon showed a peak of ~ 300 DMRs, and the gene body presented an average of 250 DMRs. In CHH, the distribution was relatively constant with 150 DMRs, except in the start positions where a depression of ~ 50 DMRs was located.

### Sexual versus apomictic analysis

**Fig. 6:**
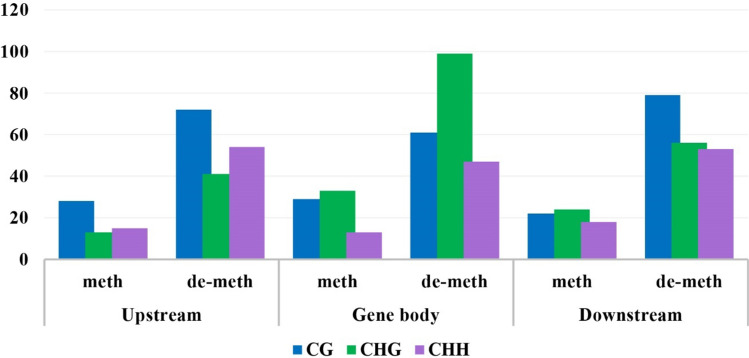
DMGs methylated or de-methylated in upstream, gene body and downstream regions in common in the comparisons between the sexual and the apomictic genotypes (vicVStan, vicVSbah, and vicVSt93)

The differentially methylated and de-methylated genes in common in the comparisons between sexual and apomictic genotypes (i.e., vicVSbah, vicVStan, vicVSt93) were analyzed in detail since they could shed light on the role of methylation in the regulation of the reproductive pathways (Fig. 6, Figure S4, Table S6). Methylated and de-methylated genes were computed using Victoria as a reference (i.e., methylated genes are methylated in the sexual genotype and de-methylated in the apomictic ones and vice versa). ANOVA test showed that the number of de-methylated genes in the sexual genotype was significantly higher than the methylated ones and predominantly in the CG context, except for the gene body, in which CHG presented more DMGs. Gene ontology enrichment analysis in CG, CHG, and CHH contexts in the upstream, gene body, and downstream regions showed terms such as DNA replication, DNA repair, helicase activity, and ubiquitin pathway, which were previously associated with reproduction in *Eragrostis curvula* (Carballo et al. 2021a, b) (Figure S5). The region being methylated/de-methylated is crucial as it can have a differential effect on gene expression (He et al. 2020, 2022; Bewick and Schmitz 2017). Even though there is no universal rule, methylation in upstream regions generally represses gene expression. Here, we observed that all the genes related to the ubiquitin pathway, such as *BPM2, AFR, E3 ubiquitin-protein ligase,* and *GA20OX2,* were de-methylated in upstream regions in the sexual genotype. The same behavior was observed for the helicase genes *RECQL2* and *RECQ4A*. These genes prevent recombination and repair DNA during meiosis (Serra et al. 2018; Kobbe et al. 2008).

Contrary to what happens at the upstream region, methylation in the gene body is associated with an increase in gene expression (Zhang et al. 2006; Bewick and Schmitz 2017). In this region, the genes related to ubiquitination were also found de-methylated. Other genes previously associated with reproduction and methylation, such as *EMB2758* and *NRPD2A*, were also found in this analysis differentially methylated in the gene body (Tzafrir et al. 2004; Zhang et al. 2021). However, one of the most interesting genes methylated in the gene body was *SPO11-1,* which is involved in recombination during meiosis and is part of the MiMe mutant used to transform mitosis into meiosis (Grelon et al. 2001; d'Erfurth et al. 2009; Miulet et al. 2016). Another gene de-methylated in the sexual genotype in the gene body was *FAR2*. This gene is essential for pollen development and was found downregulated in sexual genotypes when compared with apomictic ones in *Limonium* spp. (Caperta et al. 2023).

Even though methylated genes in the downstream region suggest a decreased gene expression, there is evidence for and against this mechanism (He et al. 2022). As in the upstream region, the genes related to the ubiquitin pathway were mostly de-methylated here. Other genes related to replication, such as *MCM6* and *CDT1A* were found to be methylated and de-methylated, respectively (Bell and Dutta 2002; Castellano et al. 2004).

### Methylation changes in diploidized and tetraploidized genotypes

**Fig. 7:**
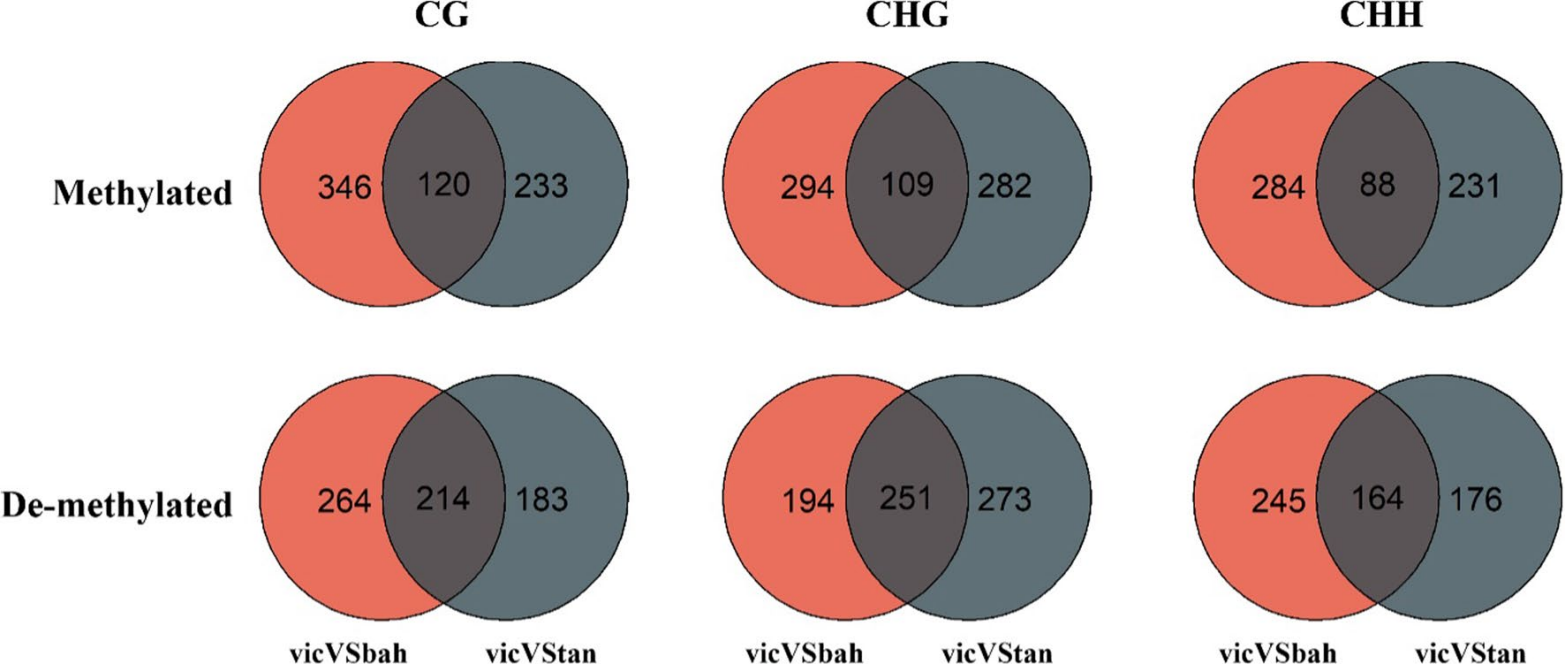
Venn diagram representing the number of methylated and de-methylated genes in the comparisons vicVSbah (red) and vicVStan (black). The intersection represents genes methylated in Victoria and de-methylated in Bahiense and Tanganyika in the upper panel and vice versa in the lower panel. The number of de-methylated genes in common between the two comparisons (intersections) was higher than the methylated ones

The comparisons vicVStan and vicVSbah were also particularly analyzed since Victoria was obtained from the diploidization of Tanganyika INTA and Bahiense from the tetraploidization of Victoria (Fig. 1). To assess the effects of methylation changes during diploidization and tetraploidization, DMGs detected in common in the comparisons vicVStan and vicVSbah were analyzed. The proportion of genes de-methylated in the upstream region observed in common (i.e., genes de-methylated in the diploid Victoria and methylated in both tetraploid genotypes, Bahiense and Tanganyika INTA) was higher than the methylated in the three contexts (Fig. 7). This suggests that many genes that were de-methylated during the diploidization process were methylated again after tetraploidization. In the three regions, upstream gene body and downstream, many DMGs were found involved in the ubiquitin pathway, such as *E3 ubiquitin-protein ligase*, *RING protein*, *BPM2*, *BPM4,* and *SKP1-like*, among others (Table S7).

Genes associated with reproduction and/or ploidy were found methylated and de-methylated in these comparisons. In the upstream region, *RECQ4A* was found de-methylated in the diploid genotype. *RECQ4A* is involved in the repression of crossovers during meiosis (Serra et al. 2018). On the other hand, the genes *SUVH5*, *HAP2,* and *NRPD2A* were found methylated in the same region. *SUVH5* is a histone methyltransferase (Rajakumara et al. 2011), *HAP2* is only expressed in haploid pollen and it is required for pollen tube guidance and fertilization (Valansi et al. 2017), and *NRPD2A* is involved in transcriptional gene silencing through RdDM pathway (Kanno et al. 2008).

In the gene body, the genes *GASA4*, *E2F1*, *CAS1*, *ULT1*, *FAR2*, *FDM1*, *SEC15B*, *OSB1*, *MEE44*, *GSL11*, *RECQ4A,* and *PC*-*MYB1* were found to be de-methylated, and *SPO11-1* and *FK* were methylated in the diploid genotype and de-methylated in the polyploids. These genes are related to cell division or reproduction; however, *PC-MYB1* and *SPO11-1* are directly involved in these pathways. *PC-MYB1* is required to maintain the ploidy level, and its mutation induces deregulation of the cell cycle and increase of the ploidy level (Haga et al. 2011). *SPO11-1* is a key meiotic gene that initiates recombination and controls double-strand breaks and crossovers (Xue et al. 2018; Vrielynck et al. 2016). As in the upstream region, *NRPD2A* was found methylated in the gene body, reinforcing that methylation can be self-regulated since *NRPD2A* is involved in the RdDM pathway. In the same way, *SUVH5*, a methyltransferase that mediates non-CG methylation, was also found methylated in the gene body.

In the downstream region, the genes *AT5G64030-like*, *AT3G11760-like*, *CDT1A*, *HAG1*, *VPS9A,* and *FDM1* were found de-methylated, and *GSL12*, *EDM2*, *MCM6*, *NRPD2B,* and *ETG1* were methylated in the diploid genotype. Interestingly, *CDT1A* promotes polyploidization and is involved in the coordination of cell division (Brasil et al. 2017). Another gene related to ploidy and cell division is *MCM6,* which is essential to undergo a single round of replications, and it is also involved in the initiation of cell elongation at the beginning of meiosis (Vinay et al. 2023). The gene *ETG1* is associated with the MCM complex and required for sister chromatid cohesion, meaning that methylation probably regulates the MCM complex at different levels in the pathway (Schubert and Shaw 2011).

## Discussion

This work investigated the differentially methylated genes associated with reproduction and ploidy changes in recently diploidized and tetraploidized genotypes of *E. curvula* with a similar genetic background (Cardone et al. 2006). To do this, we used the MCSeEd technique to analyze differentially methylated positions, regions, and genes in plants of the related genotypes Tanganyika INTA (4 × apo), Bahiense (4 × apo), and Victoria (2 × sex) and the unrelated genotype TUNS9355 (6 × apo).

Differentially methylated and de-methylated positions were analyzed in all the CG, CHG, and CHH contexts. The genotypes were compared all against each other: vicVSbah, vicVStan, vicVSt93, bahVStan, bahVSt93, and tanVSt93. The number of DMPs between genotypes across the three DNA contexts was homogeneous for all the comparisons (Fig. 2A, Table S3). In previous works, using the same technique to compare tetraploid genotypes of *E. curvula* with different reproductive modes, it was found that CG and CHG had similar total numbers of DMPs in all the comparisons. At the same time, the total number of DMPs in the CHH context showed an increased number (Carballo et al. 2021a, b). In drought-stressed and control plants of *Zea mays*, MCSeEd detected more DMPs in CHG and CHH than in CG in stressed plants (Marconi et al. 2019). Fewer DMPs in CG means that this context is conserved after alterations such as drought stress, reproductive mode, and ploidy level. This is also consistent with what was found in *Oryza sativa,* comparing diploid and tetraploid genotypes (Zhang et al. 2015). In our study, the number of de-methylated positions was, in general, higher than the number of methylated ones in the six comparisons except for tanVSt93 and bahVStan in which a higher number of methylated positions were observed in CHG and CHH (tanVSt93) and CG (bahVStan). Thus, in most of the cases, higher ploidy levels correlate directly with methylation levels. This pattern in which overall methylation levels are higher in polyploids was also found in other species, like in the model species *Arabidopsis thaliana* when compared with its tetraploid counterpart *A. arenosa* (Jiang et al. 2021). In the same way, the number of methylated sites in the autotetraploid *O. sativa* was higher than in diploids (Zhang et al. 2015; Rao et al. 2023).

The comparisons between the related genotypes vicVSbah and vicVStan showed a lower number of DMRs than the other comparisons, suggesting that even though they have differences in terms of ploidy and reproduction, the overall methylation landscape is maintained. Although most of the DMRs are within intergenic regions, the MCSeEd method properly reduces the genome's complexity since the number of intergenic/repetitive regions does not overwhelm the analysis and produces good resolution over the regulatory regions (Fig. 3). Using the same technique, a similar distribution in CG, CHG, and CHH contexts was also observed in *Z. mays* and *E. curvula* (Marconi et al. 2019; Carballo et al. 2021a).

The DMGs were divided into upstream, gene body, and downstream regions because the impact of methylation varies depending on which gene position is affected (Bewick and Schmitz 2017; He et al. 2020, 2022). In the upstream region, methylation changes were mainly located in the CG context, in the gene body were found in CHG, and in the downstream region. However, the distribution was homogeneous in the three contexts, more differences were observed in CG (Fig. 4). The distribution of the DMRs across the genes was similar to the one previously found in *E. curvula* (Carballo et al. 2021a). The area surrounding the start and stop codons showed more DMRs in the CG context (Fig. 5), probably, repressing or promoting gene expression if it is methylated or de-methylated, respectively (Wang et al. 2020). In CHG, a peak of DMRs was found around the stop codon, while in CHH, a depression was observed around the start codon, followed by an increase of methylation in the gene body. Contrary to what happens upstream and downstream, gene body methylation increases gene expression (Muyle et al. 2022a; Zilberman et al. 2007).

To identify methylated/de-methylated genes involved in ploidy changes and reproduction, we focused on the DMGs shared in different comparisons. To identify genes related to reproduction, the sexual genotype was compared against the apomictic ones (vicVSbah, vicVStan, and vicVSt93). To find genes involved in ploidy changes, we took advantage of the synthetic diploidized (Victoria) and tetraploidized (Bahiense) genotypes derived from the natural tetraploid Tanganyika INTA.

In the sexual versus apomictic comparisons, many genes related to the ubiquitin pathway were found differentially methylated in the upstream, gene body, and downstream regions. The regulation of apomixis through this pathway was also mentioned in different species (Galla et al. 2017; Rodrigo et al. 2017; Selva et al. 2020; Carballo et al. 2021a). In this study, the genes related to the ubiquitin pathway were mainly de-methylated in all the regions in the sexual genotype (Table S6). Key meiotic genes such as RECQL2, RECQ4A, and SPO11-1 were also found to be differentially methylated through these comparisons. For example, the *RECQ4A* gene, a DNA helicase that limits meiotic recombination and its mutation, increases the number of crossovers. *RECQL2* increases genomic stability, preventing non-productive recombination (Kobbe et al. 2008; Röhrig et al. 2018; Serra et al. 2018). *SPO11-1* mediates the initiation of DNA double-strand breaks and was used successfully together with *REC8* and *OSD1* to transform mitosis into meiosis (d'Erfurth et al. 2009; Mieulet et al. 2016; Xue et al. 2018). Since *RECQL2* and *RECQ4A* were found de-methylated in the upstream region and *SPO11-1* methylated in the gene body, it seems that these genes are being expressed in the sexual genotype and repressed in the apomictic ones, meaning that the meiotic pathway is being negatively regulated at different levels in apomictic genotypes. The most plausible hypothesis is that the repression of *SPO11-1* induces the decrease of double-stranded breaks, whereas the double-strand break repair machinery accomplished by *RECQL2* and *RECQ4A* is disturbed. Regulation of DNA crossovers during meiosis through this pathway was also suggested previously, showing a negative correlation between methylation and recombination in *A. thaliana* (Fernandes et al. 2018). Genes associated with cell division were also found differentially methylated in the downstream region in these comparisons, like *MCM6* and *CDT1A* which are part of the pre-replication complex responsible for the initiation of replication and prevent extra rounds of DNA replication (Brasil et al. 2017). *MCM6* was associated with gynoecium sex expression in different species and was involved in sexual differentiation (Vinay et al. 2023). Recently, it was found that in *A. thaliana, CDT1A* is expressed in pollen sperm cells before fertilization (Voichek et al. 2023; He et al. 2023). Interestingly, mutation of *CDT1A* produces genotypes that, after two rounds of mitosis, produce abnormal embryo sacs with only four nuclei, which is exactly what happens in the normal *E. curvula* apomictic embryo sac development (Crane 2001; Domenichini et al. 2012). The *CHX19* gene is methylated in the downstream region and expressed in pollen grains and tubes (Padmanaban et al. 2017). Mutation of this gene, together with *CHX17* and *CHX18,* produces male and female abnormalities. One of these abnormalities is a single event of egg or central cell fertilization, but not both (Padmanaban et al. 2017). This phenomenon could be related to the fact that in *E. curvula* apomictic genotypes, only the central cell is fertilized, while the egg cell develops a parthenogenetic embryo without fertilization (Carballo et al. 2021a, b).

Another mechanism that could increase methylation in polyploid genotypes is the dosage compensation. Apomictic species are vastly polyploids and contain a dominant non-recombinant hemizygous region/s associated with their components. For instance, Pennisetum, Taraxacum, and Paspalum it was described as a single region linked to parthenogenesis (Ozias-Akins et al. 1998; Hojsgaard et al. 2011; Van Dijk et al. 2020; Underwood et al. 2022). In *E. curvula*, a single region was also linked to apomeiosis (Zappacosta et al. 2019). Many of the characteristics associated with these regions, such as large rearrangements, accumulation of repetitive elements, and suppression of recombination, agree with the early stage of sex chromosomes (Charlesworth 2015). As in other species, the increase in methylation could be related to the dosage compensation effect, in which gene dosage does not correlate with the gene expression level (Muyle et al. 2022b). Another effect closely related to the dosage compensation that could be associated to the increase/decrease of methylation is the parent-of-origin effect. Apomictic embryos originate exclusively by the mitotic replication of the maternal genome (Crane 2001), while sexual embryos result from the fusion of the male (sperm cell) and female (egg cell) gamete. This imbalance of genome dosage was also found to be regulated by methylation, suggesting that the changes observed in *E. curvula* could be related to both parent-of-origin and dosage compensation effects (Adams et al. 2000; Duszynska et al. 2013).

Ploidy changes were assessed through the comparisons vicVStan and vicVSbah. Here, it was demonstrated that many genes that changed their status during diploidization recovered the original state after tetraploidization, meaning that there is a typical methylation landscape related to each ploidy level, as was stated previously in this species (Ochogavía et al. 2009). As in the methylation assessment with contrasting reproductive modes, here, the genes *RECQ4A, SPO11-1,* and *MCM6* were also identified as differentially methylated. In this comparison, genes specifically associated with ploidy were also found. In particular, the mutation of *PC-MYB1* induces tetraploidization in *A. thaliana* (Haga et al. 2011). Here, we found this gene de-methylated in the gene body in the diploid and methylated in the tetraploids, probably maintaining the polyploid level in tetraploids and avoiding the tetraploidization of diploids. *CDT1A* is also another gene that could be related to ploidy changes. This gene was found de-methylated in the downstream region in diploids. Overexpression of this gene in *A. thaliana* promotes endoreduplication, which is associated with increased ploidy (Raynaud et al. 2005). Since the effect of methylation in the downstream regions is not completely understood, and either repression or promotion of this gene could lead to different hypotheses, it is not clear how this gene could regulate ploidy levels/changes (He et al. 2022). The following genes involved in cell division that might also have a function associated with increasing, decrease or maintenance of ploidy levels were identified in these comparisons: *PEL1*, *GSL04*, *AT5G50340-like*, *E2F1*, *SEC15B*, *OSB*, *GSL11*, *FK*, *AT3G11760-like*, *VPS9A*, *ETG1,* and *AT3G11760-like*. As in other studies, genes related to methylation, such as *FDM1*, *SUVH5*, *NRPD2A,* and *EDM2,* were also found differentially methylated here, suggesting that this pathway could be auto-regulated (Lei et al. 2015; Williams et al. 2015; Zhang et al. 2018; Carballo et al. 2021a).

In conclusion, here we present a comprehensive view of the methylation changes that occur across genotypes with different reproductive modes and ploidy levels and its possible effects on these characteristics. Some genetic changes introduced by the diploidization and tetraploidization processes were probably involved in the changes of the reproductive mode. For instance, the loss of a genomic portion in Victoria could have introduced rearrangements altering the reproductive pathway. However, the epigenetic landscape necessary to maintain the apomictic behavior in both Tanganyika INTA and Bahiense should also be reflected in the MCSeEd analysis in the vicVStan and vicVSbah comparisons. Even more, it is hypothesized that the genes involved in the sexual pathway are repressed by epigenetic mechanisms in apomictic genotypes (Albertini et al. 2019). Some of the genes found here, like the ones involved in meiosis, agree with this theory. The comparisons between sexual and apomictic genotypes showed a general increase of methylation in apomictic versus sexual genotypes. Also, it was possible to observe methylation changes affecting genes involved in the three components of apomixis: apomeiosis, parthenogenesis, and pseudogamy. Regarding ploidy, a general increase in methylation was associated with increases in ploidy, and genes related to changes and maintenance of ploidy were also found to be differentially methylated. Even more, many genes related to embryo sac development and ploidy found differentially methylated here, in concord with previous works. In contrast, others were not reported, opening new candidate genes and pathways potentially involved in these traits.

### Supplementary Information

Below is the link to the electronic supplementary material.

Supplementary file1 (XLSX 90 KB)

Supplementary file2 (PDF 1082 KB)

## Data Availability

The datasets presented in this study can be found in online repositories: https://www.ncbi.nlm.nih.gov/, BioProject: PRJNA988943.
