# Supplementary material for: Differentially methylated genes involved in reproduction and ploidy levels in recent diploidized and tetraploidized Eragrostis curvula genotypes
Source: Plant Reprod. 2023 Dec 6;37(2):133–45. doi: 10.1007/s00497-023-00490-7 (PMC11180019; doi:10.1007/s00497-023-00490-7)
Supplement: Supplementary file 2 — Supplementary file2 (PDF 1082 KB) [file 497_2023_490_MOESM2_ESM.pdf]

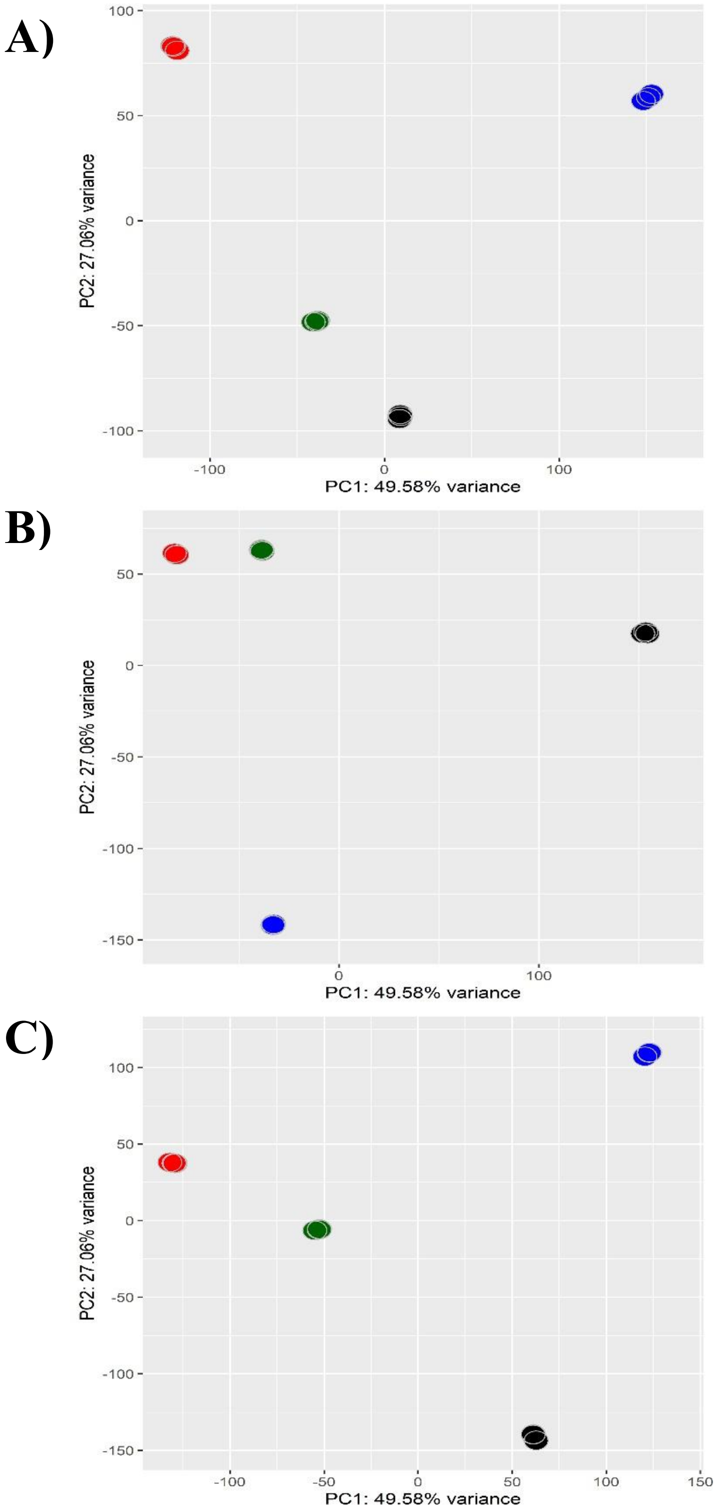

Figure S1: PCA analysis for the CG (A), CHG (B) and CHH (C) contexts. In the X and Y axis are shown the PC1 and PC2 respectively with its percentage of variance. Red circles represent Victoria samples, green circles Bahiense, blue circles Tanganyika INTA and black circles TUNS9355.

A)

B)

C)

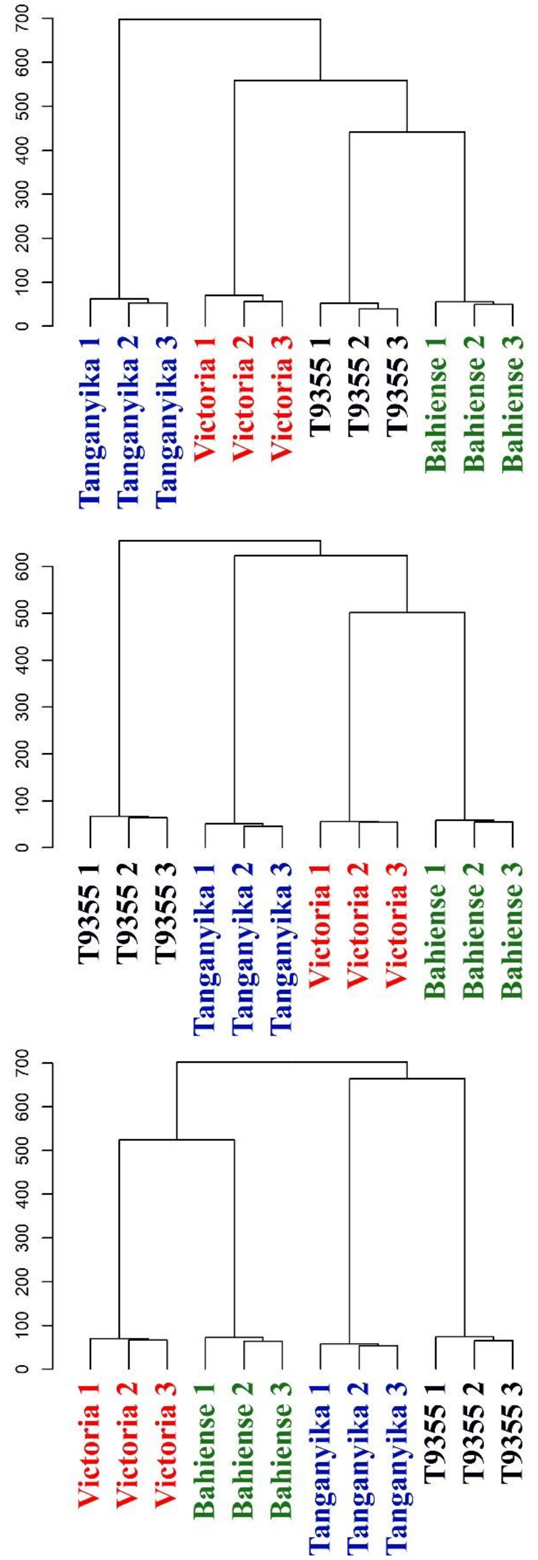

Figure S2: Samples and genotype clustering for Victoria, Bahiense, Tanganyika INTA, and TUNS9355 for CG (A), CHG (B) and CHH (C) contexts.

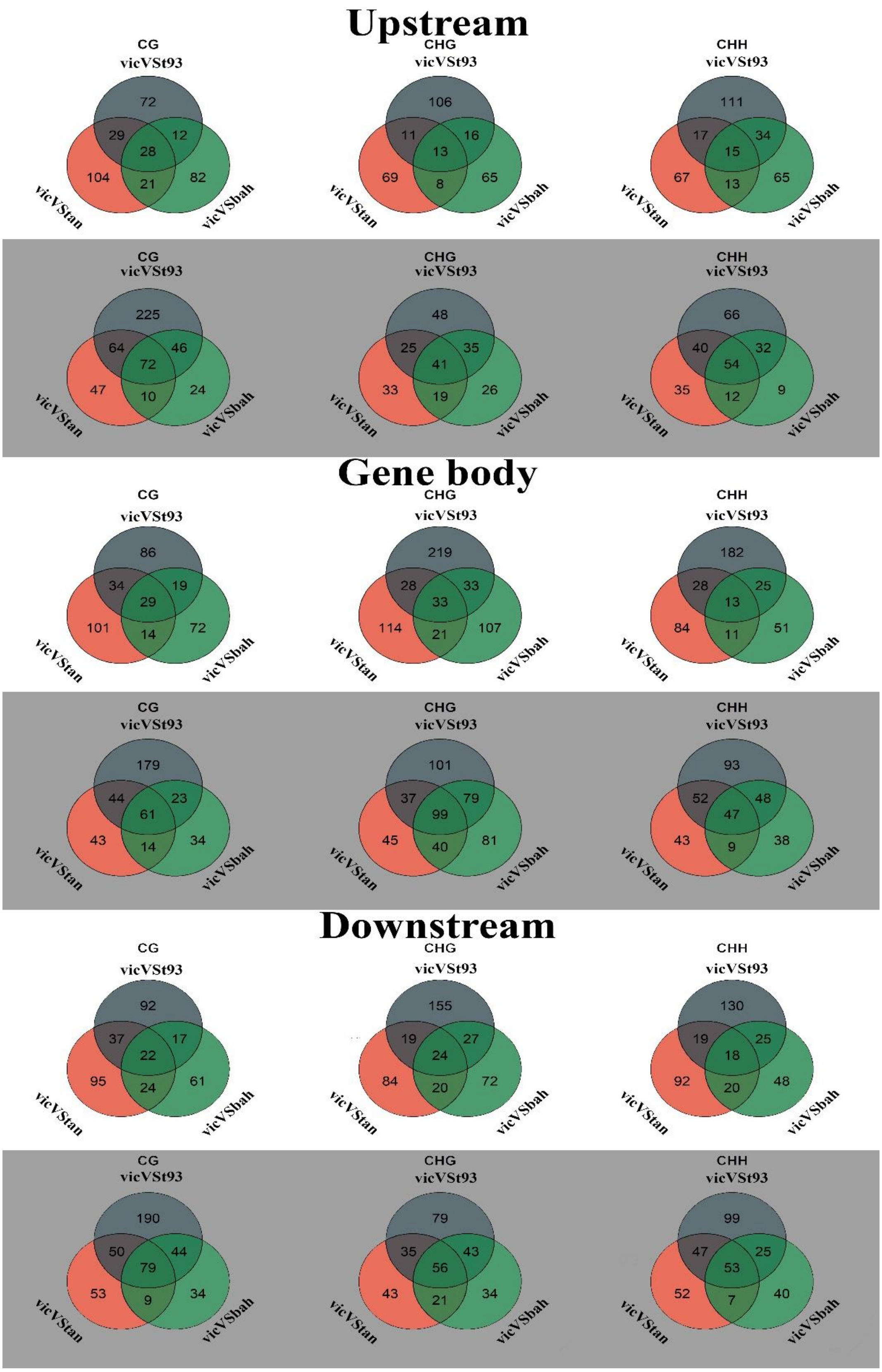

Figure S3: Venn diagram with the number of methylated (white areas) and de-methylated (gray areas) genes for the comparisons vicVSbah, vicVStan, and vicVSt93 in the upstream, gene body and downstream region in the CG, CHG, and CHH methylation contexts.

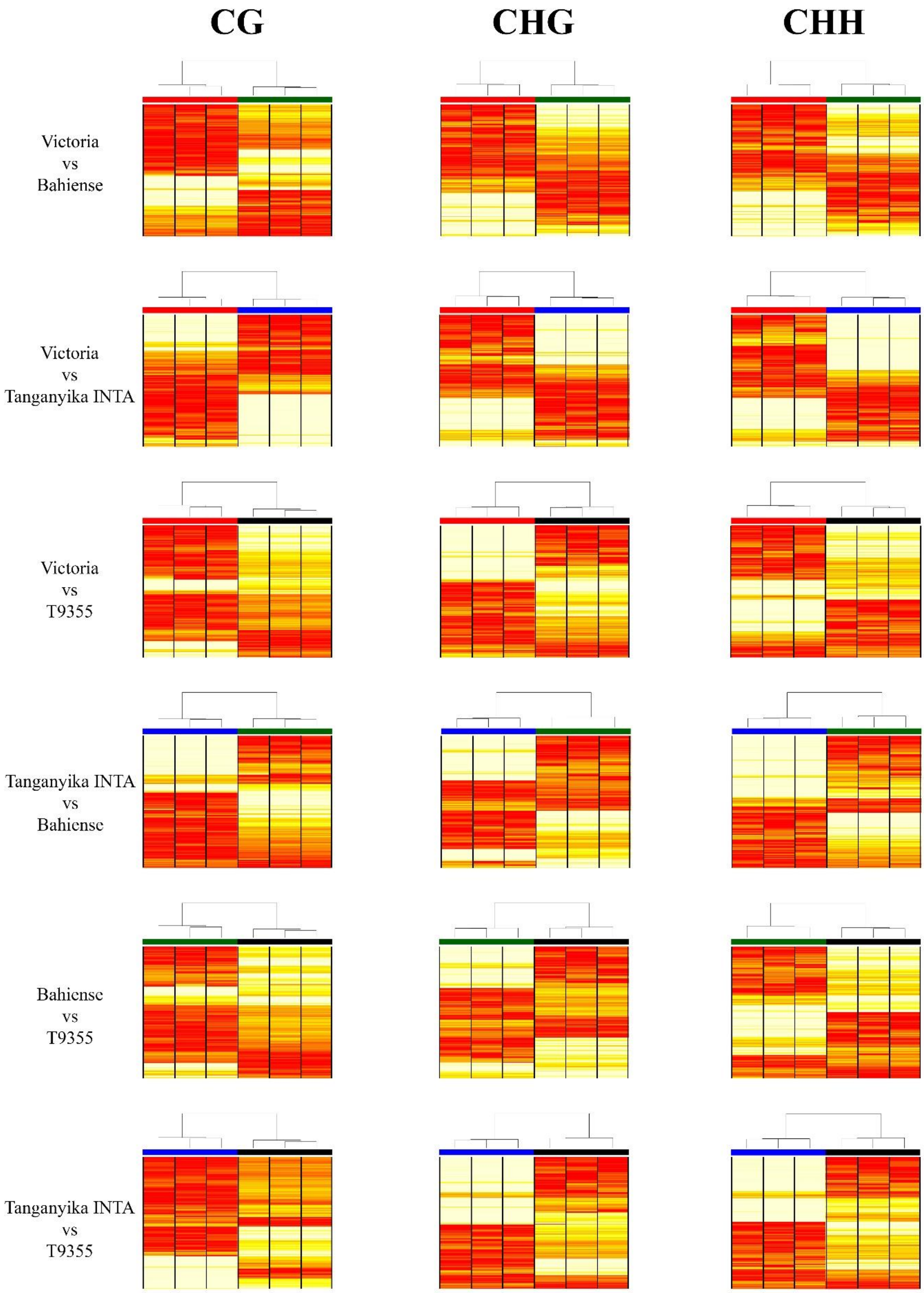

Figure S4: Heat map of DMPs within DMRs for the CG, CHG, and CHH contexts for the five comparisons indicated on the left of the plot. Victoria samples are highlighted with red bars at the top of the heatmap, Bahiense with green bars, Tanganyika INTA with blue bars, and TUNS9355 with black bars.

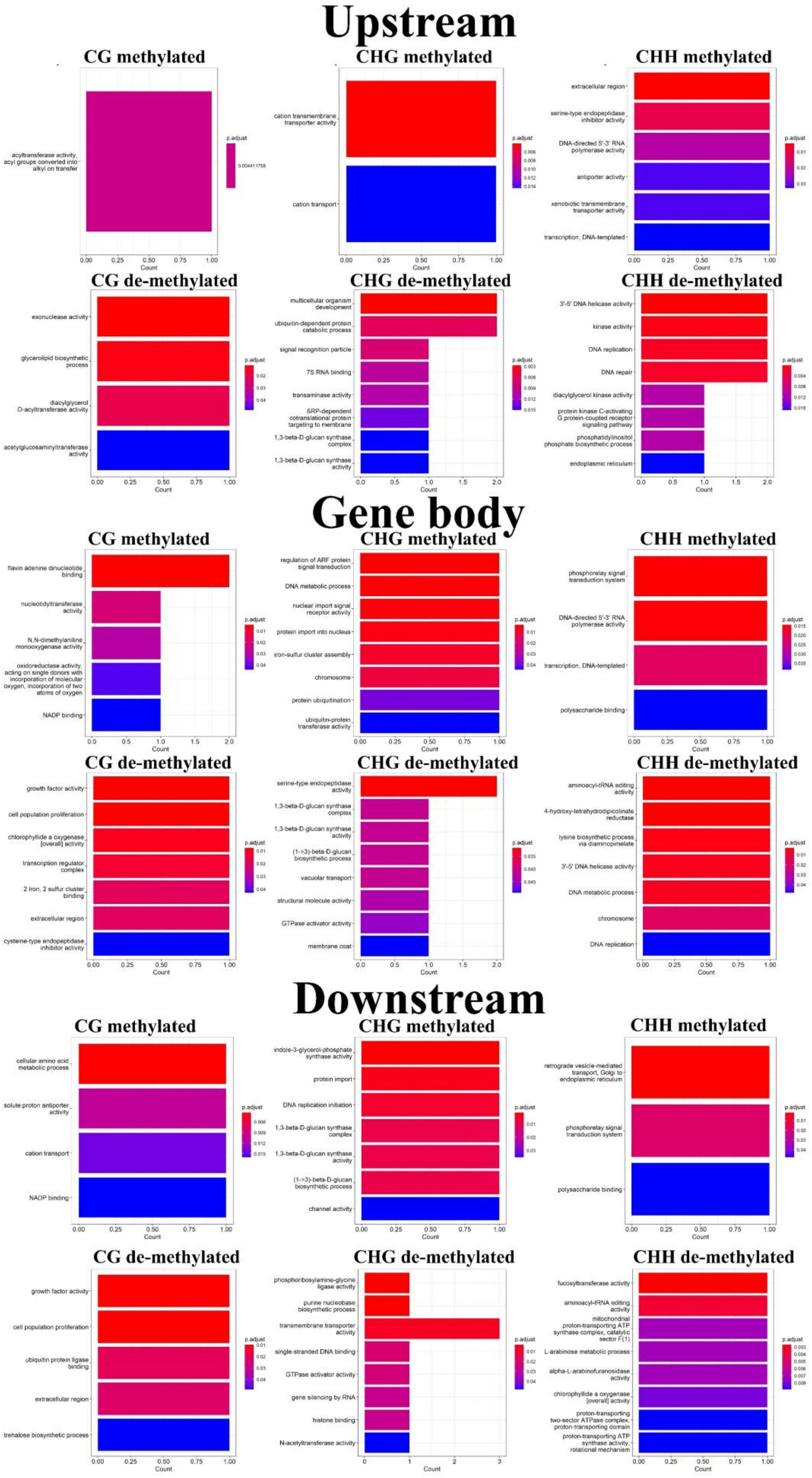
Upstream
CG methylated
acyltransferase activity, acyl groups converted into alkyl on transfer
p.adjust
0.004411758
Count
CHG methylated
cation transmembrane transporter activity
cation transport
p.adjust
Count
CHH methylated
extracellular region
serine-type endopeptidase inhibitor activity
DNA-directed 5'-3' RNA polymerase activity
antiporter activity
xenobiotic transmembrane transporter activity
transcription, DNA-templated
p.adjust
Count
CG de-methylated
exonuclease activity
glycerolipid biosynthetic process
diacylglycerol O-acyltransferase activity
acetylglucosaminyltransferase activity
p.adjust
Count
CHG de-methylated
multicellular organism development
ubiquitin-dependent protein catabolic process
signal recognition particle
7S RNA binding
transaminase activity
SRP-dependent cotranslational protein targeting to membrane
1,3-beta-D-glucan synthase complex
1,3-beta-D-glucan synthase activity
p.adjust
Count
CHH de-methylated
3'-5' DNA helicase activity
kinase activity
DNA replication
DNA repair
diacylglycerol kinase activity
protein kinase C-activating G protein-coupled receptor signaling pathway
phosphatidylinositol phosphate biosynthetic process
endoplasmic reticulum
p.adjust
Count
Gene body
CG methylated
flavin adenine dinucleotide binding
nucleotidyltransferase activity
N,N-dimethylaniline monooxygenase activity
oxidoreductase activity, acting on single donors with incorporation of molecular oxygen, incorporation of two atoms of oxygen
NADP binding
p.adjust
Count
CHG methylated
regulation of ARF protein signal transduction
DNA metabolic process
nuclear import signal receptor activity
protein import into nucleus
iron-sulfur cluster assembly
chromosome
protein ubiquitination
ubiquitin-protein transferase activity
p.adjust
Count
CHH methylated
phosphorelay signal transduction system
DNA-directed 5'-3' RNA polymerase activity
transcription, DNA-templated
polysaccharide binding
p.adjust
Count
CG de-methylated
growth factor activity
cell population proliferation
chlorophyllide a oxygenase [overall] activity
transcription regulator complex
2 iron, 2 sulfur cluster binding
extracellular region
cysteine-type endopeptidase inhibitor activity
p.adjust
Count
CHG de-methylated
serine-type endopeptidase activity
1,3-beta-D-glucan synthase complex
1,3-beta-D-glucan synthase activity
(1->3)-beta-D-glucan biosynthetic process
vacuolar transport
structural molecule activity
GTPase activator activity
membrane coat
p.adjust
Count
CHH de-methylated
aminoacyl-tRNA editing activity
4-hydroxy-tetrahydrodipicolinate reductase
lysine biosynthetic process via diaminopimelate
3'-5' DNA helicase activity
DNA metabolic process
chromosome
DNA replication
p.adjust
Count
Downstream
CG methylated
cellular amino acid metabolic process
solute:proton antiporter activity
cation transport
NADP binding
p.adjust
Count
CHG methylated
indole-3-glycerol-phosphate synthase activity
protein import
DNA replication initiation
1,3-beta-D-glucan synthase complex
1,3-beta-D-glucan synthase activity
(1->3)-beta-D-glucan biosynthetic process
channel activity
p.adjust
Count
CHH methylated
retrograde vesicle-mediated transport, Golgi to endoplasmic reticulum
phosphorelay signal transduction system
polysaccharide binding
p.adjust
Count
CG de-methylated
growth factor activity
cell population proliferation
ubiquitin protein ligase binding
extracellular region
trehalose biosynthetic process
p.adjust
Count
CHG de-methylated
phosphoribosylamine-glycine ligase activity
purine nucleobase biosynthetic process
transmembrane transporter activity
single-stranded DNA binding
GTPase activator activity
gene silencing by RNA
histone binding
N-acetyltransferase activity
p.adjust
Count
CHH de-methylated
fucosyltransferase activity
aminoacyl-tRNA editing activity
mitochondrial proton-transporting ATP synthase complex, catalytic sector F(1)
L-arabinose metabolic process
alpha-L-arabinofuranosidase activity
chlorophyllide a oxygenase [overall] activity
proton-transporting two-sector ATPase complex, proton-transporting domain
proton-transporting ATP synthase activity, rotational mechanism
p.adjust
Count

Figure S5: Differentially GO enrichment analysis over the methylated and de-methylated genes shared between de sexual vs. apomictic comparison in upstream, gene body, and downstream region
